# Draft Genome Sequence of *Epilithonimonas* sp. FP211-J200, Isolated from an Outbreak Episode on a Rainbow Trout (*Oncorhynchus mykiss*) Farm

**DOI:** 10.1128/genomeA.00819-17

**Published:** 2017-09-14

**Authors:** Manuel Ayala, Cristopher Segovia, Rodrigo Rojas, Claudio Miranda, Javier Santander

**Affiliations:** a Marine Microbial Pathogenesis and Vaccinology Laboratory, Department of Ocean Sciences, Memorial University of Newfoundland, St. John’s, Canada; b Faculty of Biological Sciences, Universidad Nacional de San Marcos, Lima, Perú; c Faculty of Sciences, Universidad Mayor, Huechuraba, Chile; d Departamento de Acuicultura, Universidad Católica del Norte, Coquimbo, Chile; e Centro AquaPacífico, Coquimbo, Chile

## Abstract

Here, we report the draft genome sequence of *Epilithonimonas* sp. FP211-J200, isolated from rainbow trout head kidney cells. The size of the genome is 4,110,772 bp, with a G+C content of 37.1%. The *Epilithonimonas* sp. FP211-J200 genome has genes related to tetracycline and β-lactam resistance. This is the first reported *Epilithonimonas* species genome isolated from a fish host.

## GENOME ANNOUNCEMENT

The genus *Epilithonimonas* (1) belongs to the phylum *Bacteriodetes*, family *Flavobacteriaceae*, which also includes the *Flavobacterium* and *Chryseobacterium* genera. Currently, five species of *Epilithonimonas* have been described (2), including *E. tenax* (1), *E. ginsengisoli* (3), *E. lactis* (4), *E. xixisoli* (5), and *E. psychrotolerans* (6). *Epilithonimonas* sp. strains have been isolated from different environments, including soil (3, 6), freshwater (1, 5), and milk (4). Members of the genus *Epilithonimonas* are chemoorganotrophs and most likely play a role in natural carbon cycles in low-salinity ecosystems, such as soil and freshwater (7). However, associations of *Epilithonimonas* spp. with animals like fish are unknown. Here, we report the draft genome sequence of *Epilithonimonas* sp. FP211-J200, a yellow-pigmented strain isolated from rainbow trout (*Oncorhynchus mykiss*) head kidney cells during a flavobacteriosis outbreak in a freshwater aquaculture facility at X Region, Chile.

*Epilithonimonas* sp. FP211-J200 was routinely grown in tryptone-yeast extract-salt (TYES) (8) with aeration (180 rpm) at 28°C. The genomic DNA was extracted according to Wilson (9) and purified using silica (10). Sequencing was performed using the next-generation sequencer (NGS) Illumina MiSeq platform (Universidad Mayor, Center for Genomics and Bioinformatics, Huechuraba, Chile) and paired-end libraries. Low-quality sequences were examined by FastQC version 0.10.1 (11). The sequences were trimmed and assembled using the CLC Genomics Workbench 9.0.1 (Qiagen) *de novo* tool, resulting in 83 contigs over 1 kb, with an *N*_50_ value of 98,567 bp. The total length of the draft genome of the *Epilithonimonas* sp. FP211-J200 was 4,110,772 bp, with a G+C content of 37.01%.

The assembled sequences were annotated by the National Center for Biotechnology Information (NCBI) Prokaryotic Genome Annotation Pipeline (PGAP [https://www.ncbi.nlm.nih.gov/genome/annotation_prok/]). The tRNA genes were detected by tRNAscan-SE version 1.3 (12) and the rRNA with RNAmmer (13). A total of 3,882 coding sequences (CDSs), 88 pseudogenes, 1 complete rRNA operon (5S-16S-23S), 41 tRNAs, and 2 noncoding RNAs (ncRNAs) were predicted by the pipeline.

Genes encoding proteins for resistance to antibiotics were identified using the Comprehensive Antibiotic Resistance Database (CARD) (14). *Epilithonimonas* sp. FP211-J200 presented genes potentially responsible for antibiotic resistance. We found three types of multidrug efflux transporters, including *acrB*, *mexD*, and *adeB* (15). Also, we found the flavin-dependent monooxygenase *tetX* gene responsible for resistance to all clinical relevant tetracyclines (16). Additionally, we found the β-lactamase TLA-2, also present in *Chryseobacterium gleum* (17), and the metallo-β-lactamase GOB-1, also present in *Chryseobacterium meningoseptica* (18). Also, we identified the *crfA* gene, which is related to florfenicol resistance.

We identified several genes related to iron acquisition and virulence, including hemolysin, hemolysin III, ferritin, ferric siderophore ABC transporter substrate-binding protein, ferredoxin, hemin receptor, and the Fur transcriptional regulator, suggesting that *Epilithonimonas* sp. FP211-J200 might have pathogenesis potential.

Phylogenetic reconstruction using 16S rRNA showed that the *Epilithonimonas* sp. FP211-J200 strain is closely related to the genus *Chryseobacterium*. *In silico* DNA-DNA hybridization (http://cbrc.kaust.edu.sa/dna_hybridization/index.html) showed that strain FP211-J200 is different from other *Epilithonimonas* spp. and *Chryseobacterium* spp. The strain most closely related to FP211-J200 was *E. ginsengisoli*. This is the first reported *Epilithonimonas* sp. genome isolated from a fish host.

### Accession number(s).

The whole-genome shotgun project (BioProject PRJNA310285) has been deposited at DDBJ/EMBL/GenBank under the accession number LSHB00000000. The version described in this paper is version LSHB01000000.
